# Protective ileostomy increased the incidence of rectal stenosis after anterior resection for rectal cancer

**DOI:** 10.1186/s13014-022-02031-4

**Published:** 2022-05-12

**Authors:** Hui Zhang, Shanshan Li, Xin Jin, Xian Wu, Zhiyuan Zhang, Lijun Shen, Juefeng Wan, Yan Wang, Yaqi Wang, Wang Yang, Menglong Zhou, Jing Zhang, Tao Lv, Yun Deng, Fan Xia, Zhen Zhang

**Affiliations:** 1 Department of Radiation Oncology, Fudan University Shanghai Cancer Center, 270 Dong An Road, Shanghai, 200032 China; 2 Department of Oncology, Shanghai Medical College, Fudan University, Shanghai, 200032 China; 3 Shanghai Key Laboratory of Radiation Oncology, Shanghai, 200032 China; 4 Department of Oncology, Zoucheng People’s Hospital, Zoucheng, 273500 Shandong China; 5 Cancer Institute, Fudan University Shanghai Cancer Center, Shanghai, 200032 China

**Keywords:** Rectal cancer, Stenosis, Preventive ileostomy, Radiotherapy

## Abstract

**Background:**

In most of the views, rectal stenosis after anterior resection for rectal cancer results from pelvic radiotherapy. However, patients without receiving radiotherapy also suffer stenosis. In this study, we evaluated the factors associated with rectal stenosis after anterior rectal resection (ARR).

**Methods:**

We conducted a retrospective study with ARR patients who underwent neoadjuvant chemoradiotherapy and the patients without radiotherapy. Patients who received watch and wait strategy with a clinical complete response after chemoradiotherapy were also included. Patients with colonoscopy follow-up were included for further analyses; 439 patients who underwent neoadjuvant chemoradiotherapy; 545 patients who received ARR without radiotherapy and 33 patients who received watch and wait strategy. Stenosis was diagnosed when a 12-mm diameter colonoscopy could not be passed through the rectum. Univariate and multivariate logistic regression analyses were performed to identify variables associated with rectal stenosis.

**Results:**

According to the multivariate analysis in patients receiving ARR, both protective stoma and preoperative radiotherapy affected the occurrence of stenosis, with the odds ratios (ORs) of 3.375 and 2.251, respectively. According to the multivariate analysis, a preventive ileostomy was the only factor associated with stenosis both in patients receiving preoperative radiotherapy and without radiotherapy. Non-reversal ileostomy and long time between ileostomy and restoration increased the possibility of stenosis. In 33 patients who received watch and wait strategy, only one patient (3%) experienced stenosis.

**Conclusion:**

Both surgery and radiotherapy are risk factors for rectal stenosis in rectal cancer patients. Compared to preoperative radiotherapy, a protective ileostomy is a more critical factor associated with rectal stenosis.

**Supplementary Information:**

The online version contains supplementary material available at 10.1186/s13014-022-02031-4.

## Background

Colorectal cancer (CRC) is the third most common malignancy among men and women [1], and rectal cancer accounts for up to 50% of all colorectal cancers. Neoadjuvant chemoradiotherapy followed by total mesorectal excision (TME) has become the standard care for T3-4 and node-positive tumors in rectal cancer [2]. However, after TME surgery, a certain number of patients will experience anastomotic complications, including anastomotic leakage and stenosis. Anastomotic leakage is a feared complication after anterior resection as it increases postoperative mortality. A temporary ileostomy created at the time of surgery may reduce the potential leak [3]. Rectal stenosis represents a challenging complication after rectal resection and can become a long-term complication among rectal cancer patients, it will seriously affect the quality of life in high-grade stenosis patients. The pathophysiology and contributing factors have only been partially understood. Postoperative anastomotic leakage and radiotherapy have been reported as predisposing factors [4–6]. In most of the views, pelvic radiotherapy is the most important reason for rectal stenosis. However, patients without receiving radiotherapy also suffer stenosis. Few studies published to date have analyzed the incidence of rectal stenosis after ARR in rectal cancer patients. This study evaluated the incidence and risk factors of rectal stenosis so that effective measurements can be adopted to mitigate the event.

## Patients and methods

### Patients and treatment

All of the consecutive patients diagnosed with rectal adenocarcinoma and who received anterior rectal resection (ARR) at Fudan University Shanghai Cancer Center between January 2006 and December 2018 were retrospectively studied, including receiving neoadjuvant chemoradiotherapy and without receiving radiotherapy. The exclusion criteria were short-course radiotherapy (5 Gy × 5 fractions), no colonoscopy follow-up, abdominoperineal resection (APR), and palliative resection. Neoadjuvant chemoradiotherapy was indicated for patients with lesions of the lower and middle rectum as T3 or T4 and for those lymph nodes suspected of being metastatic. The intensity-modulated radiation therapy (IMRT) technique was performed with a photon beam of 6-MV energy. The planned treatment dose of radiation was 50 Gy in 25 fractions (2 Gy/fraction), 5 fractions/week. The RTOG contouring atlas was referenced for clinical tumor volume (CTV) contouring [7]. Concurrent chemotherapy was conducted in 97% of all the patients, and concurrent chemotherapy regimens are capecitabine, oxaliplatin and irinotecan (Additional file 1: Table S1). Capecitabine was administered concurrently with radiotherapy on radiation days. Patients received capecitabine 825 mg/m^2^ bid orally on radiation days for the capecitabine regimen. Approximately ten years ago, we conducted a phase 2 clinical trial that explored oxaliplatin's role with capecitabine concurrent with radiotherapy in advanced rectal cancer patients (unpublished data). For the oxaliplatin regimen, patients received oxaliplatin 50 mg/m^2^ every week, concurrent with capecitabine 625 mg/m^2^ bid orally on radiation days. Recently, irinotecan and capecitabine concurrently with radiotherapy showed excellent effects in our phase 1, 2 [8, 9] and phase 3 trials [10]. Patients received capecitabine (625 mg/m^2^, bid) orally along with weekly irinotecan for 5 weeks according to the UGT1A1/28 genotype. The weekly irinotecan dose was 80 mg/m^2^ in patients with the *1*1 genotype and 65 mg/m^2^ in those with the *1*28 genotype. Surgery was undertaken following the principles of TME for patients. 439 patients received neoadjuvant chemoradiotherapy following ARR and 545 patients underwent ARR without preoperative or postoperative radiotherapy.

We also retrospectively studied 33 patients who received non-operative strategy after achieving clinical complete response (cCR) between 2015 and 2020.

The potential risk factors analyzed for the stenosis were gender, age, tumor distance from the anal margin (≤ 5 cm or > 5 cm), tumor staging (cT and cN status were assessed by magnetic resonance imaging (MRI)), body mass index (BMI), smoking, drinking, hypertension, diabetes, radiotherapy dose (RT dose), concurrent chemotherapy, pattern of surgery (open or laparoscopic), occurrence of anastomotic fistula, protective ileostomy, tumor regression grade (TRG) score, and RT response (pCR[pathologic complete response]/almost pCR or poor response). The work was in accordance with The Code of Ethics of the World Medical Association (Declaration of Helsinki) and was approved by the hospital's Medical Ethics Committee.

### Evaluation of rectal stenosis

Rectal stenosis is difficult to diagnose and grade, colonoscopy is commonly used to evaluate stenosis. Patients were encouraged to engage in a regular follow-up after the operation. Colonoscopy was performed before the restoration of protective ileostomy or the first year after surgery, and every 2–3 years after that, or in case of new symptoms or suspected relapse. Anastomotic or rectal stenosis was diagnosed when a 12-mm diameter colonoscopy could not be passed through the rectum. We found that stenosis was mainly located above the anastomoses, and not at the site of anastomoses. Colonoscopy was performed on 439 patients, who underwent neoadjuvant chemoradiotherapy followed by ARR. A group of 545 patients who received ARR without radiotherapy and underwent colonoscopy examination was formed to explore whether other factors affect rectal stenosis regardless of radiotherapy. Further, 33 patients who received nonoperative management when exhibiting cCR after neo-adjuvant chemoradiotherapy was analyzed for stenosis.

### Statistical analysis

Statistical analysis was conducted using SPSS software. We analyzed the categorical variables using the chi-square test and the quantitative ones with the Student's *t*-test (mean and standard deviation [SD]). Initially, we performed a univariate analysis for each independent variable. Then, the candidates who had a *p*-value ≤ 0.05 were considered for the multivariate model. *P* < 0.05 was considered statistically significant and marked with asterisks (*).

## Results

### Both protective ileostomy and preoperative radiotherapy were related to stenosis in patients receiving anterior rectal resection

**Fig. 1:**
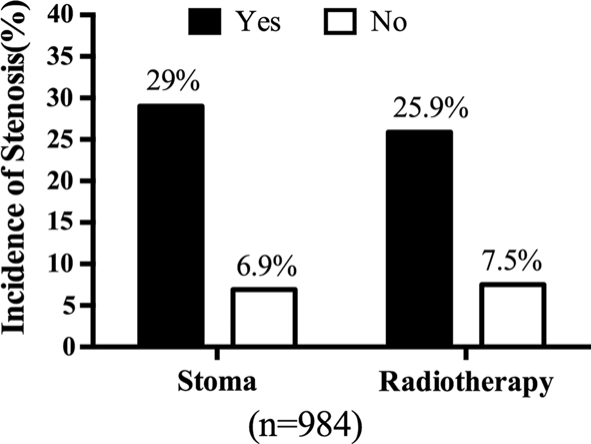
The incidence of stenosis in patients received preventive ileostoma or preoperative radiotherapy. 114 diagnosed with stenoses in patients with stoma, and 279 did not, the rate of stenoses was 114/(114 + 279) = 29%. And the rate of stenoses was 6.9% in no stoma patients. The rate of stenoses was 26.0% (114/(114 + 324)) and 7.5% (41/(41 + 504)) in radiotherapy and non-radiotherapy groups, respectively

Combining the data of preoperative radiotherapy and non-radiotherapy, there were 155 patients presenting stenosis. Gender, tumor location, smoking, surgery pattern, protective ileostomy, and radiotherapy were significantly different between the stenosis and non-stenosis groups (*P* < 0.05*, Additional file 1: Table S2). After performing multivariate analysis, only protective ileostomy and radiotherapy were the significant factors (Table 1). In the stenosis group, 73.5% of patients conducted protective ileostomy and 33.7% in the non-stenosis group. Protective ileostomy increased 3.375 the risk of stenosis. In patients with stenosis, 72.8% received preoperative radiotherapy, and the proportion was 38.4% in the non-stenosis group. Preoperative radiotherapy increased 2.251 the risk of stenosis. In 393 patients with an ileostomy, there were 29.0% (114/393) patients with stenosis and 6.9% (41/591) patients suffering stenosis without ileostomy (Fig. 1). In 424 patients with preoperative radiotherapy, 25.9% (114/439) experienced stenosis, in contrast with 7.5% (41/545) in non-radiotherapy patients.

**Table 1:** Multivariate logistic regression analysis of the association between the factors and stenosis in all non-radiotherapy and preoperative radiotherapy patients

Variables	Stenosis, N = 155	No stenosis, N = 829	OR	95% CI	*P* value
Sex, n (%)			1.133	0.738–1.742	0.568
Male	108 (69.7)	493 (59.5)			
Female	47 (30.3)	336 (40.5)			
Tumor location, n (%)			0.900	0.600–1.351	0.611
≤ 5 cm to anus	58 (37.4)	182 (22.0)			
> 5 cm to anus	97 (62.6)	647 (78.0)			
Smoking, n (%)			1.572	0.996–2.480	0.052
Yes	45 (29.0)	158 (19.1)			
No	110 (71.0)	671 (80.9)			
Surgery, n (%)			1.039	0.689–1.568	0.864
Open	92 (59.4)	620 (74.8)			
Laparoscopic	63 (40.6)	209 (25.2)			
Stoma, n (%)			3.375	2.083–5.470	0.000*
Yes	114 (73.5)	279 (33.7)			
No	41 (26.5)	550 (66.3)			
Radiotherapy, n (%)			2.251	1.440–3.519	0.000*
Yes	110 (72.8)	314 (38.4)			
No	41 (27.2)	504 (61.6)			

*OR* odds ratio; *CI* confidence interval

^*^Statistically significant difference

### Protective ileostomy was the only independent factor related to stenosis in patients with preoperative chemoradiotherapy

We identified 439 patients treated with neoadjuvant chemoradiotherapy followed by ARR and with follow-up data on colonoscopy. All the patients received intensity-modulated RT (IMRT) and TME surgery, and the total planned dose of 50 Gy in 25 fractions.

In patients with preoperative radiotherapy (Additional file 1: Table S2), 25.9% (114/439) patients experienced stenosis; the mean ages were 55.0 years (SD: ± 10.5) and 54.7 years (SD: ± 10.7) in the stenosis and non-stenosis group, respectively (*p* > 0.05). By performing a chi-square test (Additional file 1: Table S2), comorbidities, such as diabetes and hypertension, were not different between the stenosis and non-stenosis groups. The tumor location, BMI, history of drinking and smoking, cT stage, cN stage, RT dose, concurrent chemotherapy, the pattern of surgery, the occurrence of leakage, and TRG score were not significantly different between the two groups. Males, smoking and patients who underwent preventive ileostomy were more likely to suffer from stenosis. In 114 patients with stenosis, 82 (71.9%) were male, and 32 (28.1%) were female; the proportion of males was much higher in the stenosis group (*P* = 0.047*). More males (29.1%; 82/282) suffered from stenosis than females (20.4%; 32/157). More patients (29.8%) had a history of smoking in the stenosis group than in the non-stenosis group (19.4%; *P* = 0.026*). A protective ileostomy was the most significantly different factor; 80.7% of patients received ileostomy in the stenosis group, while only 64.6% of patients had an ileostomy in the non-stenosis group (*P* = 0.001*). After performing multivariate analysis (Table 2), the only statistically significant predictor of stenosis was the presence of ileostomy (*P* = 0.003*), and ileostomy increased the risk of stenosis by 2.2. Among the 302 patients receiving ileostomy, 92 (30.5%) patients experienced stenosis, which is much higher than the 22 (16.1%) patients with stenosis without ileostomy.

**Table 2:** Multivariate logistic regression analysis of the association between the factors and stenosis in preoperative radiotherapy

Variables	Stenosis	No stenosis	OR	95% CI	*P*-value
	N = 41	N = 504			
Sex, n (%)			1.307	0.784–2.180	0.305
Female	32 (28.1%)	125 (38.5%)			
Male	82 (71.9%)	200 (61.5%)			
Smoking, n (%)			1.520	0.891–2.591	0.124
No	80 (70.2)	262 (80.6)			
Yes	34 (29.8)	63 (19.4)			
Stoma, n (%)			2.200	1.306–3.705	0.003*
No	22 (19.3)	115 (35.4)			
Yes	92 (80.7)	210 (64.6)			

*OR* odds ratio; *CI* confidence interval

^*^Statistically significant difference

**Fig. 2:**
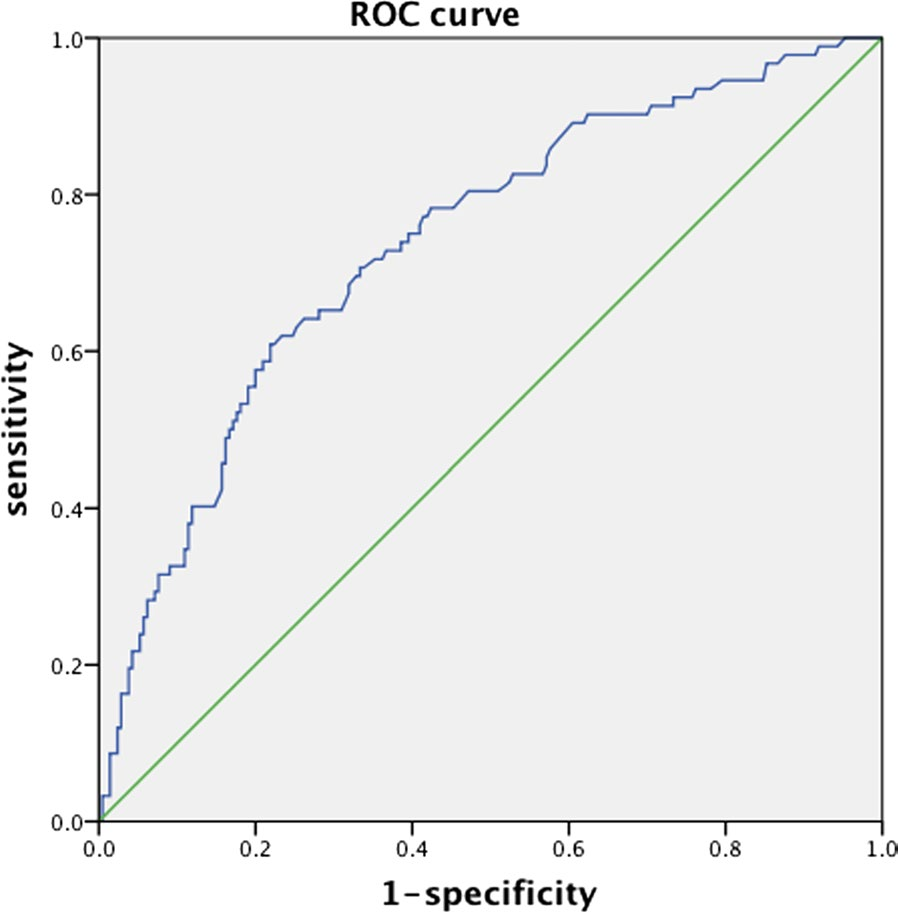
The ROC curve for the cut-off point of the interval between ileostomy and restoration to predicting ractal stenosis

Of the 302 patients who had a protective ileostomy, 259 (85.8%) patients had the ileostomy closed, and 53 (17.5%) remained with the stoma. Only 65.2% patients received a restoration in the stenosis group, and 90% in the non-stenosis group (*P* = 0.001*). Fifty-three patients did not receive restoration, 17 patients because of stenosis, 13 patients because of metastasis, 3 patients because of the presence of both stenosis and metastasis, 5 for leakage, 2 for relapse, and 14 for unknown reasons. To study if the interval between radiotherapy and ARR affected stenosis, we also analyzed the time from the end of radiotherapy to ARR. The result did not show a significant difference; the mean time in the stenosis and non-stenosis groups was 63.359 ± 17.035 days and 60.667 ± 23.102 days, respectively (*P* = 0.316). Furthermore, we calculated the interval between ARR and restoration, and anylzed the cut-off value using ROC (the receiving operator characteristic) curve, it showed that the cut-off value was about six months (Fig. 2). Then, we divided the patients into those with less than six months from stomy to restoration and those that did not receive restoration within six months. Patients who did not receive restoration were defined as not receive restoration within six months. In the stenosis group, 81.5% of patients received restoration more than 6 months after ileostomy, and this proportion was only 52.4% in the non-stenosis group (Table 3).

**Table 3:** The parameters of stoma and stenosis in patients received stoma

	Stenosis	No stenosis	*P*-value
	N = 92	N = 210	
Restored, n (%)			0.001*
No	32 (34.8)	21 (10.0)	
Yes	60 (65.2)	189 (90.0)	
Time from end of RT to surgery (d)			0.316
Mean ± SD	63.359 ± 17.035	60.667 ± 23.102	
Time from stoma to restoration, n (%)			0.001*
≤ 6 m	17 (18.5)	100 (47.6)	
> 6 m	75 (81.5)	110 (52.4)	

^*^Statistically significant difference

### Protective ileostomy was the only factor related to stenosis in patients without radiotherapy

In most of the views, stenosis is the late side effect of radiotherapy. To explain this problem, we also studied if patients without radiotherapy will present with stenosis. Five hundred forty-five patients who did not receive radiotherapy but had colonoscopy follow-up was included. The incidence rate of stenosis was 7.5% (41/545). By performing a chi-square test (Additional file 1: Table S3), the clinical characteristics were not significantly different between the stenosis and non-stenosis groups for gender, smoking, drinking, hypertension, diabetes, pT stage, pN stage, and the occurrence of leakage. However, age, tumor location, surgery pattern and protective ileostomy were imbalanced between the two groups. Older age, tumor in the lower location, and laparoscopic surgery increased the incidence of stenosis. After performing the multivariate analysis (Table 4), only protective ileostomy was statistically significant for stenosis (*P* = 0.001*). In the stenosis group, 80.7% of the patients received protective ileostomy, while only 64.6% in the non-stenosis group did. The odd ratio of stomy was 2.533, and a similar result was obtained for the preoperative radiotherapy patients.

**Table 4:** Multivariate logistic regression analysis of the association between the factors and stenosis in non-preoperative radiotherapy

Variables	Stenosis	No stenosis	OR	95% CI	*P* value
Age (y)					
Mean ± SD	61.5 ± 10.8	57.8 ± 10	0.999	0.979–1.020	0.936
Tumor location, n (%)			0.972	0.622–1.518	0.899
≤ 5 cm to anus	11 (26.8%)	59 (11.7%)			
> 5 cm to anus	30 (73.2%)	445 (88.3%)			
Stoma, n (%)			2.533	1.438–4.463	0.001*
No	22 (19.3)	115 (35.4)			
Yes	92 (80.7)	210 (64.6)			
Surgery, n (%)			0.795	0.493–1.281	0.345
Open	24 (58.5)	417 (82.7)			
Laparoscopic	17 (41.5)	87 (17.3)			

*OR* odds ratio; *CI* confidence interval

^*^Statistically significant difference

### Fewer stenosis appear in patients receiving non-operative management

Non-operative management for lower rectal cancer patients with a cCR after neoadjuvant chemoradiotherapy is a valuable alternative for rectal resection in recent years. In our center, watch and wait strategy was used in lower rectal cancer patients from 2015. To explain if stenosis will appear in patients who received non-operative management when achieve cCR after neoadjuvant chemoradiotherapy. Thirty-three patients who received non-operative management and had colonoscopy follow-up were retrospectively analyzed. The mean coloscopy follow time was more than 2 years. Of the 33 patients who received non-operative treatment, only one experienced stenosis. The rate of stenosis was much lower in non-operative patients than in ARR patients (Table 5).

**Table 5:** Clinical characteristics of the patients who received non-operative treatment

	Stenosis	No stenosis
	N = 1	N = 32
Age (y)		
Mean ± SD	36	56.8 ± 9.6
Sex, n (%)		
Male	1 (100)	25 (78.1)
Female	0 (0)	7 (21.9)
Tumor location (distance to anus, cm)		
Mean ± SD	1	3.6 ± 1.5
Smoking, n (%)		
Yes	0 (0)	1 (3.1)
No	1 (100)	31 (96.9)
Concurrent Chemo, n (%)		
No	0	0
Capecitabine	1 (100)	5 (15.6)
Irinotecan + cape	0	25 (78.1)
Others	0	2 (6.3)
Follow-up times (m)	26.6	25.2 ± 21.6
Mean ± SD		

## Discussion

Radiotherapy is an important management among multidisciplinary treatment in rectal cancer, reducing local recurrence and increasing organ preservation. Radiotherapy also plays more important roles in immunotherapy era. However, radiotherapy is always challenged by its adverse effect, such as proctitis and stenosis. In most of the views, rectal stenosis after anterior resection for rectal cancer results from pelvic radiotherapy. However, patients without receiving radiotherapy also suffer from stenosis. Adverse effects cannot be simply attributed to radiotherapy when patients receive multidisciplinary treatments. We were interested in answering the question that which factors are associated with rectal stenosis and which is the most important factor. We studied three groups of patients to explain this issue: patients receiving neoadjuvant chemoradiotherapy followed by ARR, patients receiving ARR without neoadjuvant or adjuvant radiotherapy and patients receiving non-operative management after achieving cCR. From our data, both radiotherapy and preventive ileostomy were implicated in rectal stenosis and were independent risk factors.

It has been reported that the incidence of rectal stenosis ranges from 2 to 30% [11–14]. The incidence of stenosis was 26.0% in patients with preoperative radiotherapy and 7.5% in patients without radiotherapy at our center. The incidence of stenosis is in accordance with the reported range of 2–30%. The absence of a precise definition account for this wide range. It is high in the preoperative radiotherapy group at our center. There are some reasons to explain this finding. First, we recorded all the patients who had difficulty in passing a 12-mm colonoscopy, including asymptomatic patients. Second, radiotherapy contributed to stenosis; the high rate of stenosis was in the preoperative group. Preoperative radiotherapy is widely used at our center for patients with locally advanced disease. Finally, patients who received preoperative radiotherapy were more likely to have a lower tumor location and undergo preventive ileostomy; after that, the occurrence of stenosis was more frequent.

In most of the views, pelvic irradiation induces rectal stenosis [6]. According to our univariate analysis, males, smoking and ileostomy were significant risk factors for stenosis in the preoperative chemoradiotherapy group. Multivariate analysis confirmed the significance of ileostomy, as 30.5% of patients with ileostomy experienced stenoses, whereas the incidence was only 16.1% in the non-stomy group (*P* < 0.001). In non-radiotherapy patients, stoma was also the only independent factor in multivariate analysis (OR = 2.533, *P* = 0.001). Preventive ileostomy was the only independent factor associated with stenosis in both preoperative radiotherapy and non-radiotherapy patients in our study. Most patients with resectable tumors would undergo surgery either after neoadjuvant radio- or chemotherapy or as the first step of the treatment. The common and dangerous complication is anastomotic leakage, particularly in tumors located not far from the anal verge. Due to the fistula's high rate, most authors recommend performing a loop ileostomy for protecting anastomoses [3, 15]. The stomy will be closed several months later. Some patients will lose the chance of stoma closure for several reasons, such as distant metastasis and stenosis. Interestingly, the rate of stenosis was very low in patients with non-operative management, indicating that radiotherapy is not the main factor to rectal stenosis. In fact that radiation treatment is not so important in the pathogenesis of rectal stenosis, is also highlighted by the lack of increase of this complication when delivering higher radiotherapy doses, up to 60 Gy, with simultaneous integrated boost. [16, 17]

We found that most of the stenoses occurred above the anastomosis instead of at the anastomosis. MRI images showed that the thickness of the bilateral obturator interrus increased significantly after chemoradiotherapy compared to pretreatment [18]. Rectal stenosis may be caused by radiation-induced fibrosis of the pelvic wall soft tissue. Muscle fibrosis may restrict the movement of the rectum, and this lack of motion lead to stenosis. On the other way, pelvic nerve damage induced by surgery or radiotherapy will limit the motion of the rectum too [19]. It was revealed that male and smoking are stenosis risk factors in our univariate analysis in preoperative patients. This agrees with the results of Kim MJ and Bannura GC, i.e., that a history of heavy smoking was significantly associated with anastomotic complications, such as leakage and stricture [20, 21]. Smoking exerts a negative effect on tissue oxygen supply through several mechanisms. Ischemia at the anastomosis site can cause anastomotic leakage or stricture by impeding the healing process.

The identification of risk factors for anastomotic complications can help decrease their frequency. Potential risk factors associated with rectal stenosis are preoperative radiotherapy and preventive ileostomy. Preoperative radiotherapy is confirmed as a standard treatment for locally advanced rectal cancer because it reduces local recurrence compared with postoperative radiotherapy [11, 22]. Thus, it cannot be omitted. Preventive ileostomy reduces the occurrence of leakage. Stools were not excreted through the rectum in patients with ileostomy; pelvic fibrosis can easily form because the rectum and pelvic muscles cannot move. The longer the preventive ileostomy remains, the more easily the fibrosis and stenosis will occur. In our study, the restoration of preventive stomy for more than six months will increase the occurrence of stenosis. Early closure of the protective ileostomy and anal functional training may be essential to reduce stenosis after surgery. Patients do not receive restoration during adjuvant chemotherapy result to late closure of preventive ileostomy. According to the trial of IDEA [23], some patients with low risks need fewer chemotherapy cycles and can receive early closure of stomy. Recently, the total neoadjuvant therapy (TNT) has been showing increased pCR and outcomes [24, 25]. In this new mode of treatment, patients do not need postoperative chemotherapy or need less. Thus, preventive ileostomy can be closed early. Some anti-fibrotic medicines have been evaluated by pre-clinical studies. Several clinical trials have shown some effects on radiation-induced fibrosis or other fibrotic diseases, such as idiopathic pulmonary fibrosis [26–28]. However, these have not been widely used as a standard treatment at the clinic for radiation fibrosis. More effective and tolerable anti-fibrotic drugs should be studied for use at the clinic to reduce stenosis.

There are limitations in this study. First, this is a retrospective study, there can be a recall bias, and the symptoms associated with stenosis did not been record. A further limitation is that stenosis could not be graded according to retrospective colonoscopy reports.

## Conclusion

From our data, both radiotherapy and preventive ileostomy were implicated in rectal stenosis and were the independent risk factors, and radiotherapy is not the main factor. The restoration of the preventive stomy after more than six months will increase the occurrence of stenosis. Early closure of the protective ileostomy and anal functional training may be essential to reduce stenosis after surgery. More effective and tolerable anti-fibrotic drugs should be studied and used at the clinic to reduce stenosis.

## Supplementary Information

**Additional file 1**.Clinical characteristics of patients.

## Data Availability

The data used for analyzed during the current study are available from the corresponding author on reasonable request.

## Abbreviations

ARR: Anterior rectal resection
ORs: Odds ratios
CRC: Colorectal cancer
TME: Total mesorectal excision
APR: Abdominoperineal resection
IMRT: Intensity-modulated radiation therapy
CTV: Clinical tumor volume
cCR: Clinical complete response
BMI: Body mass index
TRG: Tumor regression grade
pCR: Pathologic complete response
SD: Standard deviation
ROC: The receiving operator characteristic
TNT: Total neoadjuvant therapy
