# Supplementary material for: Protective ileostomy increased the incidence of rectal stenosis after anterior resection for rectal cancer
Source: Radiat Oncol. 2022 May 12;17:93. doi: 10.1186/s13014-022-02031-4 (PMC9097119; doi:10.1186/s13014-022-02031-4)
Supplement: Supplementary file 1 — Additional file 1.Clinical characteristics of patients. [file 13014_2022_2031_MOESM1_ESM.docx]

**Title: Protective Ileostomy Increased the Incidence of Rectal Stenosis after Anterior Resection of Rectal Cancer**

**Supplementary material: Clinical characteristics of patients**

**Table S1** Clinical characteristics of patients underwent preoperative radiotherapy

|  | Stenosis  N =114 | No stenosis  N =325 | *P*-value |
| --- | --- | --- | --- |
| Age (y)  Mean±SD | 55.0±10.5 (22-74.0) | 54.7±10.7 (24-76) | 0.845 |
| Sex, n (%)  Male  Female | 82 (71.9%)  32 (28.1%) | 200 (61.5%)  125 (38.5%) | 0.047* |
| Tumor location, n (%)  ≤ 5cm to anus  > 5cm to anus | 47 (41.2%)  67 (58.8%) | 123 (37.8%)  202 (62.2%) | 0.524 |
| BMI  Mean±SD | 22.8±2.9 | 23.1±3.1 | 0.422 |
| Smoking, n (%)  Yes  No | 34 (29.8)  80 (70.2) | 63 (19.4)  262 (80.6) | 0.026* |
| Drinking, n (%)  Yes  No | 19 (16.7)  95 (83.3) | 46 (14.2)  279 (85.8) | 0.541 |
| Hypertension, n (%)  Yes  No | 21 (18.4)  93 (81.6) | 50 (15.4)  275 (84.6) | 0.462 |
| Diabetes, n (%)  Yes  No | 12 (10.5)  102 (89.5) | 25 (7.7)  300 (92.3) | 0.334 |
| cT stage, n (%)  2  3  4 | 3 (2.6)  91 (79.8)  20 (17.5) | 6 (1.8)  283 (87.1)  36 (11.1) | 0.170 |
| cN stage, n (%)  0  1  2  N+ | 9 (7.9)  37 (32.5)  52 (45.6)  16 (14.0) | 20 (6.2)  98 (30.2)  172 (52.9)  35 (10.8) | 0.534 |
| RT dose (Gy)  Mean±SD | 49.5±3.9 | 49.5±4.2 | 0.976 |
| Concurrent Chemo, n (%)  No  Capecitabine  Oxaliplatin  Irinotecan  Others | 9 (2.1)  144 (32.8)  104 (23.7)  180 (41.0)  2 (0.5) | 6 (1.8)  103 (31.7)  70 (21.5)  144 (44.3)  2 (0.6) | 0.125 |
| Surgery pattern, n (%)  Open  Laparoscopic | 68 (59.6)  46 (40.4) | 203 (62.5)  122 (37.5) | 0.336 |
| Leakage, n (%)  Yes  No | 7 (6.1)  107 (93.9) | 17 (5.2)  308 (94.8) | 0.811 |
| Stoma, n (%)  Yes  No | 92 (80.7)  22 (19.3) | 210 (64.6)  115 (35.4) | 0.001* |
| TRG Score, n (%)  0  1  2  3 | 22 (19.3)  30 (26.3)  46 (40.4)  16 (14.0) | 78 (24.0)  64 (19.7)  142 (43.7)  41 (12.6) | 0.407 |
| RT response, n (%)  PCR/almost PCR  Poor response | 52 (45.6)  62 (54.4) | 142 (43.7)  183 (56.3) | 0.743 |

Abbreviations: BMI= body mass index; RT= Radiotherapy; TRG= tumor regression grade; PCR= pathologically complete response

Almost PCR= TRG 0+ TRG 1.

* Statistically significant difference.

**Table** S2 Clinical characteristics of all of the patients who underwent non-radiotherapy or preoperative radiotherapy

|  | Stenosis  N=155 | No stenosis  N=829 | *P* value |
| --- | --- | --- | --- |
| Age (y)  Mean±SD | 56.7±10.9 | 56.6±10.9 | 0.924 |
| Sex, n (%)  Male  Female | 108 (69.7)  47 (30.3) | 493 (59.5)  336 (40.5) | 0.019* |
| Tumor location, n (%)  ≤ 5cm to anus  > 5cm to anus | 58 (37.4)  97 (62.6) | 182 (22.0)  647 (78.0) | 0.000* |
| Smoking, n (%)  Yes  No | 45 (29.0)  110 (71.0) | 158 (19.1)  671 (80.9) | 0.007* |
| Drinking, n (%)  Yes  No | 24 (15.5)  131 (84.5) | 91 (11.0)  738 (89.0) | 0.133 |
| Hypertension, n (%)  Yes  No | 29 (18.7)  126 (81.3) | 126 (15.2)  703 (84.8) | 0.280 |
| Diabetes, n (%)  Yes  No | 14 (9.0)  141 (91.0) | 56 (6.8)  773 (93.2) | 0.308 |
| Surgery, n (%)  Open  Laparoscopic | 92 (59.4）  63 (40.6） | 620 (74.8)  209 (25.2) | 0.000* |
| Leakage, n (%)  Yes  No | 8 (5.2)  147 (94.8) | 30 (3.6)  799 (96.4) | 0.363 |
| Stoma, n (%)  Yes  No | 114 (73.5)  41 (26.5) | 279 (33.7)  550 (66.3) | 0.000* |
| Radiotherapy, n (%)  Yes  No | 110 (72.8)  41 (27.2) | 314 (38.4)  504 (61.6) | 0.000* |

* Statistically significant difference.

**Table** S3 Clinical characteristics of patients not receive radiotherapy

|  | Stenosis  N=41 | No stenosis  N=504 | *P*-value |
| --- | --- | --- | --- |
| Age (y)  Mean±SD | 61.5±10.8 | 57.8±10 | 0.035* |
| Sex, n (%)  Male  Female | 26 (63.4)  15 (36.6) | 293 (58.1)  211 (41.9) | 0.621 |
| Tumor location, n (%)  ≤ 5cm to anus  >5cm to anus | 11 (26.8)  30 (73.2) | 59 (11.7)  445 (88.3) | 0.012* |
| Smoking, n (%)  Yes  No | 11 (26.8)  30 (73.2) | 95 (18.8)  409 (81.2) | 0.220 |
| Drinking, n (%)  Yes  No | 5 (12.2)  36 (87.8) | 45 (8.9)  459 (91.1) | 0.410 |
| Hypertension, n (%)  Yes  No | 8 (19.5)  33 (80.5) | 76 (15.1)  428 (84.9) | 0.498 |
| Diabetes, n (%)  Yes  No | 2 (4.9)  39 (95.1) | 31 (6.2)  473 (93.8) | 1.000 |
| pT stage, n (%)  0  1  2  3  4 | 0 (0)  3 (7.3)  11 (26.8)  22 (53.7)  5 (12.2) | 2 (0.4)  56 (11.1)  143 (28.4)  220 (43.7)  83 (16.5) | 0.739 |
| pN satge , n (%)  0  1  2 | 27 (65.9)  10 (24.4)  4 (9.8) | 344 (68.3)  105 (20.8)  55 (10.9) | 0.858 |
| Surgery pattern, n (%)  Open  Laparoscopic | 24 (58.5)  17 (41.5) | 417 (82.7)  87 (17.3) | 0.001* |
| Leakage, n (%)  Yes  No | 1 (2.4)  40 (97.6) | 13 (2.6)  491 (97.4) | 1.000 |
| Stoma, n (%)  Yes  No | 22 (53.7)  19 (46.3) | 69 (13.7)  435 (86.3) | 0.001* |

* Statistically significant difference.
